# A cohort study of post-COVID-19 condition across the Beta, Delta, and Omicron waves in South Africa: 6-month follow-up of hospitalized and nonhospitalized participants

**DOI:** 10.1016/j.ijid.2022.12.036

**Published:** 2023-03

**Authors:** Waasila Jassat, Caroline Mudara, Caroline Vika, Richard Welch, Tracy Arendse, Murray Dryden, Lucille Blumberg, Natalie Mayet, Stefano Tempia, Arifa Parker, Jeremy Nel, Rubeshan Perumal, Michelle J. Groome, Francesca Conradie, Norbert Ndjeka, Louise Sigfrid, Laura Merson, Cheryl Cohen

**Affiliations:** 1 National Institute for Communicable Disease, Division of the National Health Laboratory Services, Johannesburg, South Africa; 2 Right to Care, Centurion, South Africa; 3 School of Public Health, Faculty of Health Sciences, University of the Witwatersrand, Johannesburg, South Africa; 4 Divisions of General Medicine and Infectious Diseases, Faculty of Medicine and Health Sciences, Stellenbosch University and Tygerberg Hospital, Cape Town, South Africa; 5 Division of Infectious Diseases, Department of Medicine, University of the Witwatersrand, Johannesburg, South Africa; 6 Division of Pulmonology and Critical Care, Department of Medicine, University of KwaZulu-Natal, Berea, Durban, South Africa; 7 South African Medical Research Council-CAPRISA HIV/TB Pathogenesis and Treatment Research Unit, Centre for the AIDS Programme of Research in South Africa, University of KwaZulu-Natal, Durban, South Africa; 8 School of Pathology, Faculty of Health Sciences, University of Witwatersrand, Johannesburg, South Africa; 9 Clinical HIV Research Unit, Faculty of Health Sciences, University of Witwatersrand, Johannesburg, South Africa; 10 Drug-Resistant TB, TB & HIV Directorate, National Department of Health, Pretoria, South Africa and University of KwaZulu-Natal, Durban, South Africa; 11 International Severe Acute Respiratory and emerging Infections Consortium (ISARIC), Pandemic Sciences Institute, University of Oxford, Oxford, United Kingdom

**Keywords:** Long COVID, Post-COVID-19 condition, Risk factors, Variants, Omicron

## Abstract

**Objectives:**

The study aimed to describe the prevalence of and risk factors for post-COVID-19 condition (PCC).

**Methods:**

This was a prospective, longitudinal observational cohort study. Hospitalized and nonhospitalized adults were randomly selected to undergo telephone assessment at 1, 3, and 6 months. Participants were assessed using a standardized questionnaire for the evaluation of symptoms and health-related quality of life. We used negative binomial regression models to determine factors associated with the presence of ≥1 symptoms at 6 months.

**Results:**

A total of 46.7% of hospitalized and 18.5% of nonhospitalized participants experienced ≥1 symptoms at 6 months (*P* ≤0.001). Among hospitalized people living with HIV, 40.4% had persistent symptoms compared with 47.1% among participants without HIV (*P* = 0.108). The risk factors for PCC included older age, female sex, non-Black race, presence of a comorbidity, greater number of acute COVID-19 symptoms, hospitalization/COVID-19 severity, and wave period (lower risk of persistent symptoms for the Omicron compared with the Beta wave). There were no associations between self-reported vaccination status with persistent symptoms.

**Conclusion:**

The study revealed a high prevalence of persistent symptoms among South African participants at 6 months but decreased risk for PCC among participants infected during the Omicron BA.1 wave. These findings have serious implications for countries with resource-constrained health care systems.

## HIGHLIGHTS

• A total of 47% of hospitalized and 19% of nonhospitalized participants had symptoms at 6 months.
• There was no difference in persistent symptoms by HIV status.
• There was a lower risk of persistent symptoms with infection during Omicron than Beta.
• There were no associations between self-reported vaccination status with persistent symptoms.

## Introduction

The post-COVID-19 condition (PCC), as defined by the World Health Organization (WHO), “occurs in individuals with a history of probable or confirmed SARS-CoV-2 infection, usually 3 months from the onset of COVID-19, with symptoms that last for at least 2 months and cannot be explained by an alternative diagnosis” [1]. Conservative estimates are that 10-30% individuals infected with SARS-CoV-2 may be affected by PCC [2], whereas a study reported that as many as 60% of COVID-19 survivors will experience PCC at least during the first year [3]. Furthermore, one study has reported persistent symptoms up to 2 years after acute COVID-19 [4].

PCC displays a wide spectrum of clinical manifestations. Over 60 physical and psychological sequelae have been described [5]. The most common symptoms reported include fatigue, dyspnea, arthromyalgia, headache, cough, chest pain, sleep disturbance, depression/anxiety, and cognitive deficits (‘brain fog’), including loss of memory and difficulty concentrating [3,[6], [7], [8]].

Risk factors identified for PCC include female sex, ethnicity/race, comorbidities, greater number of acute COVID-19 symptoms, and severe COVID-19 disease [6,[9], [10], [11], [12], [13]]. Recent studies have suggested that the prevalence of PCC among people infected during the Omicron wave was lower than those infected in previous waves dominated by Delta and Alpha [9,[14], [15], [16]]. Some studies have also suggested that COVID-19 vaccination may reduce the risk of developing PCC [17], [18], [19]. A single study suggested that the risk of PCC appears to increase with SARS-CoV-2 reinfection [20].

Existing studies on PCC are highly heterogeneous, with varying sample sizes, including patients with different acute COVID-19 severity and time frames for analysis [21]. Many studies have not assessed risk factors and the impact of SARS-CoV-2 variant or have been conducted with a short follow-up duration. Most have been set in high-income settings [5,22].

South Africa experienced five COVID-19 waves, dominated by the D614G mutation, Beta, Delta, Omicron BA.1, and Omicron BA.4/BA.5 variants, respectively. As of September 06, 2022, over 4 million cases [23], 542,332 hospitalizations, and 104,302 deaths [24] have been reported. Nationally, 50% of adults have been fully vaccinated [25]. Recent anti-SARS-CoV-2 antibody seroprevalence surveys conducted just before the fifth wave revealed that over 90% of South Africans have immunity, the majority of whom had an antibody profile consistent with previous SARS-CoV-2 infection [26]. It is therefore likely that a large number of people could be affected by PCC in South Africa, a country with a strained public health system before the pandemic, which poses a challenge for the delivery of multidisciplinary care for individuals with PCC.

To characterize and identify risk factors for developing PCC in the population in South Africa, we established a longitudinal cohort study, following up hospitalized and nonhospitalized patients for 6 months after laboratory-confirmed SARS-CoV-2 infection. This study was led by the National Institute for Communicable Diseases, as part of a global study coordinated by the International Severe Acute Respiratory and emerging Infections Consortium (ISARIC). This is the first study in South Africa to characterize PCC up to 6 months after SARS-CoV-2 infection among hospitalized and nonhospitalized participants and the impact of acute COVID-19 severity, HIV, SARS-CoV-2 variants (Beta, Delta, and Omicron), and COVID-19 vaccination on the risk of developing PCC.

## Methods

### Study design

This was a prospective, longitudinal observational cohort study using an ISARIC open-access tool that was locally adapted to follow-up participants with laboratory-confirmed COVID-19 in South Africa [27].

### Study population and sampling

The study population included individuals aged 18 years and older, with a positive reverse transcriptase-polymerase chain reaction assay or rapid antigen test for SARS-CoV-2, who had a recorded contact number. Participants included those who had symptoms and those who were asymptomatic and tested for SARS-CoV-2 for reasons including exposure to SARS-CoV-2, for travel, and routine hospital admission screening. Four cohorts of patients were recruited (i) hospitalized Beta wave, (ii) hospitalized Delta wave, (iii) hospitalized Omicron wave, and (iv) nonhospitalized Delta wave. Nonhospitalized patients were identified through the national case line list. Hospitalized patients were identified from public and private hospitals from all provinces of South Africa through the Daily Hospital Surveillance (DATCOV) national hospital surveillance.

The sample size calculation considered a proportion of participants with ≥1 persistent symptoms of 66% at 6 months from the literature; odds ratio (OR) = 1.5 for those with severe COVID-19 to have persistent symptoms; assuming a consistent difference in the proportion of the outcome of interest at four different periods: 1, 3, 6 and 12 months; a correlation of 0.6 in outcome measurements for each individual over time; alpha 0.05 and power 80%. The upper bound of the sample size calculated using two methods was 544. We adjusted for attrition at 50%, resulting in sample size of 816 participants per cohort. However, due to the likelihood of uncontactable people due to poor recording of contact details in national surveillance systems, the possibility of greater attrition than anticipated and the need to learn more about this new evolving disease, we randomly sampled approximately 3500 patients per cohort and invited them to participate in this study by telephone. Random sampling was done using a computer-generated list of eligible participants. If selected participants were not available, they were recontacted twice until they were excluded. Verbal consent was obtained and recorded, and, where possible, assessments were conducted by trained researchers in the language of the participants’ choice. Hospitalized participants infected during Beta and Delta waves had 1-, 3-, and 6-month assessments; those hospitalized during the Omicron wave and nonhospitalized participants infected during the Delta wave had 3- and 6-month assessments.

### Measurements and instruments

A standardized case report form, developed by ISARIC, documented demographic variables, comorbidities, COVID-19 vaccination status, acute symptoms and severity, current health status, and new or persistent symptoms. The case report form contained validated tools to establish quality of life [28], dyspnea (modified Medical Research Council dyspnea scale) [29], and functioning (UN/Washington Disability Score) [30]. COVID-19 vaccination status was self-reported by participants and categorized as not vaccinated or fully vaccinated before or after the SARS-CoV-2 infection. Individuals were considered to be fully vaccinated if they received two doses of BNT162b or one dose of Ad26.COV2.S, with the most recent dose at least 14 days earlier and unvaccinated if these criteria were not met. These are the only two vaccines provided in the South African COVID-19 vaccination program).

The WHO COVID-19 clinical progression scale captured the range of clinical manifestations of patients with acute COVID-19 [31]. These scales were adapted to categorize levels of severity relevant to the in-hospital and nonhospitalized cohorts: (i) nonhospitalized asymptomatic, (ii) nonhospitalized symptomatic, (iii) hospitalized (no oxygen therapy), (iv) hospitalized (oxygen by nasal prongs or mask), (v) hospitalized (mechanical ventilation and/or intensive care unit [ICU]).

Data were entered and stored on a secure online Research Electronic Data Capture repository (REDCap, version 10.6.14, Vanderbilt University, Nashville, TN, USA) hosted by the University of Oxford on behalf of ISARIC.

### Statistical analysis

Frequencies and percentages were used to summarize categorical data, and continuous data were expressed using medians and interquartile ranges (IQR). Bivariate analysis was conducted to compare the proportions of participants with ≥1 symptoms and no symptoms by wave period of infection, hospitalization status, COVID-19 severity, and HIV status, using the Pearson chi-square test.

Negative binomial regression was implemented to assess the factors associated with ≥1 persistent symptoms at 6 months. Variables included in the multivariable model based on clinical plausibility were age, sex, ethnicity, presence of comorbid conditions (asthma, diabetes, hypertension, chronic cardiac disease, chronic kidney disease, malignancy, tuberculosis, HIV, and obesity), number of symptoms during acute infection, acute COVID-19 severity, COVID-19 vaccination status, and wave period. Variables with *P* <0.2 in the univariable analysis were included in the multivariable analysis. Manual backward elimination was implemented, and the final model selection was guided by the minimization of the Akaike information criterion or Bayesian information criterion. Statistical significance for the multivariable analysis was assessed at *P* <0.05. Statistical analyses were performed using Stata software version 16 (StataCorp Limited, College Station, TX, USA). The study was approved by the University of the Witwatersrand Human Research Ethics Committee (HREC M201150).

## Results

Of the 336,071 hospitalized and 415,155 nonhospitalized individuals eligible for inclusion during the study period, 3500 individuals were randomly selected for enrollment from each cohort (hospitalized in Beta, Delta, and Omicron waves and nonhospitalized). Of those contacted, 3334 (24.0%) hospitalized and 1351 (38.6%) nonhospitalized were able to be reached, consented to participate, and were enrolled in the study. Of the 3334 hospitalized participants recruited, 2626 (78.8%) completed 6-month assessments. Of the 1351 nonhospitalized participants recruited, 1074 (79.5%) completed 6-month assessments (Supplementary Figure S1). Study participants had a similar distribution by sex, were on average, with more sampled from the private sector, and had a higher proportion with comorbidities than the total hospitalized and nonhospitalized population (Supplementary Table S1).

### Characteristics of participants

The median age of the hospitalized participants was 49 (IQR 37-60) years; 1491 (56.8%) were females. The nonhospitalized participants’ median age was 37 [IQR 28-47] years and 641 (59.7%) were females. A pre-existing comorbidity was present in 65.1% and 32.8% of hospitalized and nonhospitalized participants, respectively (Table 1).

**Table 1:** Characteristics of hospitalized and non-hospitalized participants with and without persistent symptoms, at 6-month follow-up.

Characteristics	Hospitalized with ≥1 symptom n = 1227	Hospitalized with no symptoms n = 1399	*P*-values Hospitalized with ≥1 and no symptoms	Non-hospitalized with ≥1 symptom n = 199	Non-hospitalized with no symptoms n = 875	*P*-values Non-hospitalized with ≥1 and no symptoms
**Median age (interquartile range)**	53 [43-62]	44 [33-58]	<0.001	42 [29-50]	36 [28-46]	<0.001
**Age group**						
<40 years 40-64 years ≥65 years	220 (17.93) 774 (63.08) 233 (18.99)	577 (41.24) 613 (43.82) 209 (14.94)	<0.001	88 (44.22) 99 (49.75) 12 (6.03)	523 (59.77) 325 (37.14) 27 (3.09)	<0.001
**Sex**						
Female Male Unknown	697 (56.81) 530 (43.19)	794 (56.75) 605 (43.25)	0.979	143 (71.86) 55 (27.64) 1 (0.50)	498 (56.91) 377 (43.09)	<0.001
**Race**						
White Black Mixed race Indian Other/Asian Unknown	461 (37.57) 536 (43.68) 110 (8.96) 101 (8.23) 2 (0.16) 17 (1.39)	323 (23.09) 833 (59.54) 101 (7.22) 69 (4.93) 5 (0.36) 68 (4.86)	<0.001	81/199 (40.70) 66/199 (33.17) 34/199 (17.09) 17/199 (8.54) 1/199 (0.50) 0	246 (28.11) 480 (54.86) 109 (12.46) 39 (4.46) 1 (0.11) 0	<0.001
**Comorbidity**						
Heart Disease High blood pressure Asthma Chronic lung disease Diabetes- gestational Diabetes type I or II Kidney disease Liver disease Cancer Blood disorder Rheumatological Neurological condition Dementia HIV Tuberculosis High cholesterol Thyroid disease Depression Obesity Other	81 (6.60) 478 (38.96) 82 (6.68) 13 (1.06) - 309 (25.18) 18 (1.47) 3 (0.24) 29 (2.36) 9 (0.73) 25 (2.04) 8 (0.65) 4 (0.33) 61 (4.97) 6 (0.49) 78 (6.36) 17 (1.39) 22 (1.79) 295 (18.13) 102 (6.85)	53 (3.79) 366 (26.16) 55 (3.93) 13 (0.93) 4 (0.29) 232 (16.58) 24 (1.72) 3 (0.21) 23 (1.64) 7 (0.50) 13 (0.93) 7 (0.50) 3 (0.21) 90 (6.43) 3 (0.21) 54 (3.86) 14 (1.00) 23 (1.64) 181 (12.94) 78 (5.58)	0.001 <0.001 0.002 0.737 0.061 0.000 0.613 0.872 0.187 0.444 0.018 0.607 0.580 0.108 0.230 0.003 0.362 0.769 0.000 0.006	6/199 (3.02) 43/199 (21.61) 6/199 (3.02) 1/199 (0.50) - 20/199 (10.05) 1/199 (0.50) 1/199 (0.50) 4/199 (2.01) 0 2/199 (1.01) 1/199 (0.50) 0 4/199 (2.01) 1/199 (0.50) 7/199 (3.52) 5/199 (2.51) 8/199 (4.02) 11/199 (5.53) 20/199 (10.05)	13 (1.49) 108 (12.34) 23 (2.63) 2 (0.23) 2 (0.23) 38 (4.34) 4 (0.46) - 3 (0.34) 1 (0.11) 8 (0.91) - - 41 (4.69) - 25 (2.86) 11 (1.26) 10 (1.14) 30 (3.43) 40 (4.57)	0.140 0.001 0.761 0.509 0.500 0.001 0.932 0.036 0.008 0.633 0.904 0.036 - 0.089 0.036 0.621 0.187 0.004 0.327 0.002
**Number of comorbidities**						
No comorbidities One comorbidity Two comorbidities ≥ Three comorbidities	298 (24.29) 425 (34.64) 321 (26.16) 183 (14.91)	618 (44.17) 431 (30.81) 228 (16.30) 122 (8.72)	<0.001	107 (53.77) 52 (26.13) 27 (13.57) 13 (6.53)	615 (70.29) 177 (20.23) 65 (7.43) 18 (2.06)	<0.001
**Sector**						
Public Private	271 (22.09) 956 (77.91)	404 (28.88) 995 (71.12)	<0.001	82 (41.21) 117 (58.79)	468 (53.49) 407 (46.51)	0.002
**COVID-19 vaccination status**						
Unvaccinated Vaccinated Missing	320 (26.08) 901 (73.43) 6 (0.49)	379 (27.09) 1013 (72.41) 7 (0.50)	0.841	78 (39.20) 121 (60.80)	369 (42.17) 502 (57.37) 4 (0.46)	0.453
**Variant**						
Beta Delta Omicron BA.1	755 (61.53) 316 (25.75) 156 (12.71)	513 (36.67) 200 (14.30) 686 (49.04)	<0.001	- 199 (100.00) -	- 875 (100.00) -	

### Persistent symptoms in hospitalized versus nonhospitalized participants

**Figure 1:**
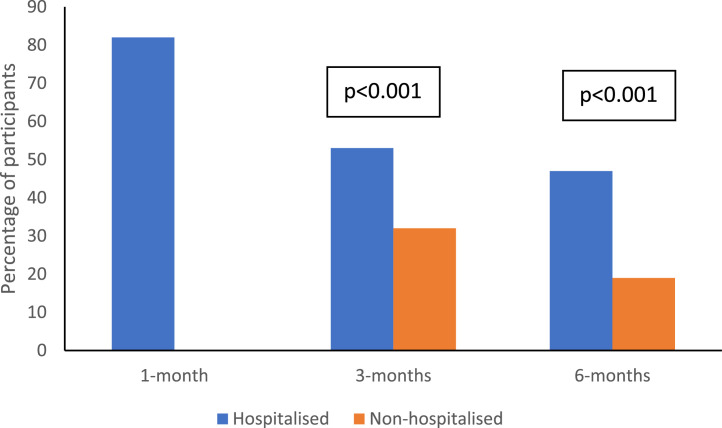
Percentage of hospitalized and non-hospitalized participants with ≥1 persistent symptoms, at 1, 3 and 6-months post-SARS-CoV-2 infection.

Among hospitalized participants, 81.7% (1932/2366) had ≥1 symptoms at 1 month, 53.5% (1518/2840) at 3 months, and 46.7% (1227/2626) at 6 months (*P* ≤0.001; Figure 1). Among nonhospitalized participants, 31.6% (427/1351) had ≥1 symptoms at 3 months, and 18.5% (199/1,074) at 6 months (*P* <0.001).

*Assessments were not conducted at 1 month for non-hospitalized participants

**Figure 2:**
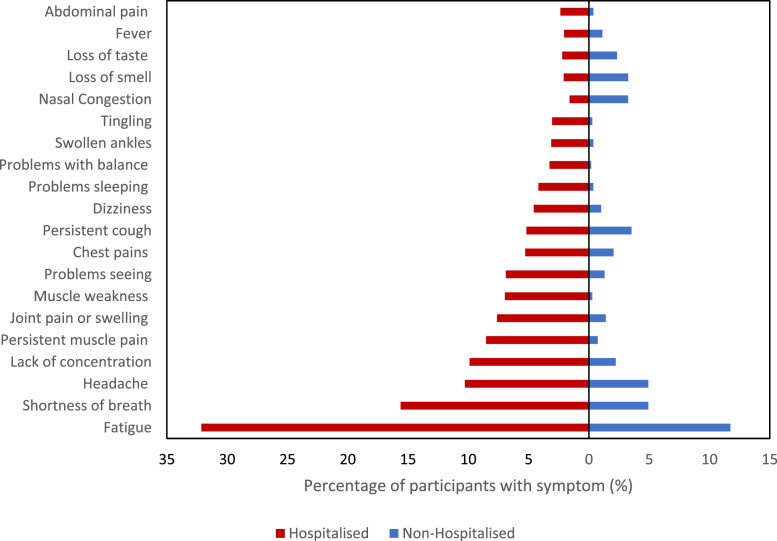
Frequency of most common symptoms among hospitalized and non-hospitalized participants at 6-months follow-up.

The most common persistent symptoms reported among hospitalized participants at 6 months (n = 2626) were fatigue (32.1%), shortness of breath (15.6%), headache (10.3%), lack of concentration (9.9%), and muscle pain (8.5%; Figure 2). The most common persistent symptoms reported among nonhospitalized participants at 6 months (n = 1074) were fatigue (11.7%), shortness of breath (4.9%), headache (4.9%), persistent cough (3.5%), loss of smell (3.3%), and nasal congestion (3.3%; Figure 2). All symptoms showed decreasing frequency over time (Supplementary Figure S2).

### Persistent symptoms by acute illness severity

Using an adapted WHO clinical severity scale, persistent symptoms at 6 months were reported among 5.6% (9/162) of nonhospitalized asymptomatic participants, 20.8% (190/912) of nonhospitalized symptomatic participants, 17.1% (105/614) of hospitalized participants (no oxygen therapy), 51.1% (335/656) of hospitalized participants (received oxygen therapy), and 58.0% (787/1356) hospitalized participants (received mechanical ventilation or treated in ICU; *P* <0.001; Supplementary Figure S3).

### Persistent symptoms among hospitalized participants by SARS-CoV-2 variant

The median age of hospitalized participants infected during the Beta wave was 52 years (IQR 41-61), Delta wave 53 years (IQR 42-63), and Omicron wave 40 years (IQR 31-56). Most were females (Beta 51.7%, Delta 52.5%, Omicron 67.1%). A pre-existing comorbidity was present in 68.2%, 77.7%, and 52.7% of participants admitted during the Beta, Delta, and Omicron waves, respectively. (Supplementary Table S1).

**Figure 3:**
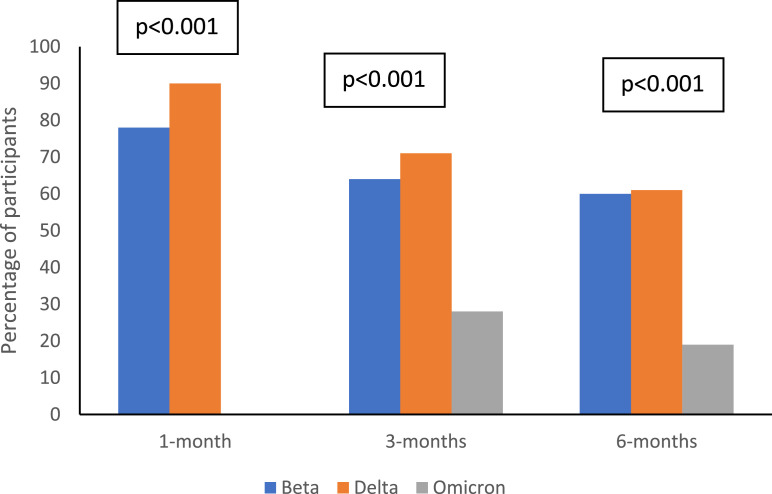
Percentage of hospitalized participants with ≥ 1 symptoms at 1, 3 and 6-month follow-up, by SARS-CoV-2 variant.

Among participants infected during the Beta wave, 77.5% (1212) experienced ≥1 symptoms at 1 month, 64.3% (779) at 3 months, and 59.5% (755) at 6 months (*P* <0.001; Figure 3). During the Delta wave, 89.7% (720) reported ≥1 symptoms at 1 month, 71.2% (469) at 3 months, and 61.2% (316) at 6 months (*P* <0.001). During the Omicron wave, 27.9% (270) reported ≥1 symptoms at 3 months and 18.5% (156) at 6 months (*P* <0.001).

*Assessments were not conducted at 1 month for participants infected during the Omicron wave.

### Persistent symptoms among people living with HIV and hospitalized participants without HIV

Among people living with HIV who were hospitalized, 43.1% (72/167) had ≥1 symptoms at 3 months and 40.4% (61/151) at 6 months (*P* = 0.624; Supplementary Figure S4). Among participants without HIV, 54.1% (1447/2674) had ≥1 symptoms at 3 months, and 47.1% (1166/2475) had ≥1 symptoms at 6 months (*P* <0.001).

### Symptom evolution among hospitalized participants

Among the 2300 hospitalized participants who completed both the 3- and 6-month follow-up assessment, the nature of symptom presentation and progression varied. Although 32.6% (749) remained symptom-free throughout the follow-up period, 16.6% (381) experienced symptoms from the acute infection for 3 months, 31.2% (718) experienced symptoms from the acute infection until 6 months, 15.5% (357) experienced new symptoms at 3-months, which continued to 6 months, and 4.7% (108) experienced new symptoms at the 6-months assessment (Supplementary Figure S5).

### Impact on quality of life among hospitalized participants

**Figure 4:**
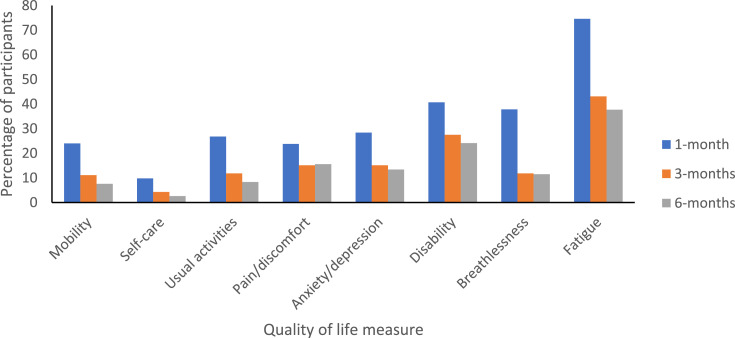
Percentage of hospitalized participants with decreased quality of life domains at 1, 3 and 6-month follow-up.

The proportion of hospitalized participants (N = 2626) who experienced reductions in quality of life domains at 6 months was 37.7% (989) for fatigue, 24.1% (633) for disability, 15.6% (410) for pain/discomfort, 13.4% (352) for anxiety/depression, 11.5% (303) for breathlessness, 8.3% (219) for usual activities, 7.6% (200) for mobility, and 2.6% (69) for self-care (Figure 4). The reported challenges with all the above quality of life measures decreased over time.

### Factors associated with persistent symptoms

On the multivariable regression, factors associated with ≥1 persistent symptoms among hospitalized and nonhospitalized participants at 6 months included age, sex, race, presence of a comorbidity, number of acute COVID-19 symptoms, COVID-19 severity, and SARS-CoV-2 variant (Table 2). There was an increased risk of persistent symptoms among older individuals aged 40-64 years (adjusted incident risk ratio [aIRR] 1.36, 95% confidence interval [CI] 1.18-1.57), and age ≥65 years (aIRR 1.28; 95% CI 1.06-1.55) compared with those aged <40 years; females (aIRR 1.24; 95% CI 1.12-1.38); and White (aIRR 1.26; 95% CI 1.11-1.38), Indian (aIRR 1.36; 95% CI 1.11-1.66), and mixed (aIRR 1.22; 95% CI 1.01-1.46) race compared with Black race. Other risk factors included the presence of a comorbidity (aIRR 1.27; 95% CI 1.12-1.43); one to three acute COVID-19 symptoms (aIRR 1.28; 95% CI 1.00-1.64) and four or more acute COVID-19 symptoms (aIRR 1.62; 95% CI 1.29-2.03) compared with no acute COVID-19 symptoms. With respect to severity of COVID-19, individuals who were nonhospitalized symptomatic (aIRR 2.32; 95% CI 1.15-4.70), hospitalized not on oxygen (aIRR 3.96; 95% CI 1.90-8.24), hospitalized on oxygen (aIRR 6.01; 95% CI 2.94-12.29), and hospitalized ventilated or treated in the ICU (aIRR 5.78; 95% CI 2.87-11.66) had a higher risk of persistent symptoms than nonhospitalized asymptomatic individuals. Individuals infected during the Omicron wave had a lower risk of persistent symptoms (aIRR 0.45; 95% CI 0.36-0.57) than those infected during the Beta wave. There were no associations between self-reported vaccination status before or after SARS-CoV-2 infection with persistent symptoms. Individual comorbidities, including hypertension, diabetes, chronic pulmonary disease, obesity, HIV, and others, were not associated with persistent symptoms on the negative binomial regression analysis.

**Table 2:** Multivariable regression of factors predicting ≥1 symptoms among hospitalized and non-hospitalized participants at 6 months after COVID-19.

Characteristic	Persistent symptoms n/N (%)	Unadjusted IRR (95% CI)	aIRR (95% CI)	*P*-value
**Age group (years)**				
<40	308/1408 (21.88)	Reference	Reference	
40-64	873/1811 (48.21)	2.20 (1.93 – 2.51)	1.36 (1.18 – 1.57)	0.0000
≥65	245/481 (50.94)	2.33 (1.96 – 2.75)	1.28 (1.06 – 1.55)	0.0095
**Sex**				
Male	585/1567 (37.33)	Reference	Reference	
Female	840/2132 (39.40)	1.06 (0.95 – 1.17)	1.24 (1.12 – 1.38)	0.0001
Unknown	1/1 (100)	2.68 (0.38 – 19.05)	3.33 (0.45 – 17.02)	0.2317
**Race**				
White	542/1111 (48.78)	1.55 (1.38 – 1.74)	1.26 (1.11 – 1.38)	0.0003
Black	602/1915 (31.44)	Reference	Reference	
Indian	118/226 (52.21)	1.66 (1.36 -2.02)	1.36 (1.11 – 1.66)	0.0026
Mixed race	144/354 (40.68)	1.29 (1.08 – 1.55)	1.22 (1.01 – 1.46)	0.0362
Other	3/9 (33.33)	1.06 (0.34 – 3.30)	1.23 (0.40 – 3.84)	0.7198
Unknown race	17/85 (20.00)	0.64 (0.39 – 1.03)	1.08 (0.66 – 1.77)	0.7653
Comorbidity				
No	405/1638 (24.73)	Reference	Reference	
Yes	1021/2062 (49.52)	2.00 (1.78 – 2.25)	1.27 (1.12 – 1.43)	0.0002
**Number of acute COVID-19 symptoms**				
0	103/671 (15.35)	Reference	Reference	
1-3	277/839 (33.02)	2.15 (1.72 – 2.70)	1.28 (1.00 – 1.64)	0.0482
≥4	1426/2190 (47.76)	3.11 (2.54 – 3.81)	1.62 (1.29 – 2.03)	0.0000
**COVID-19 severity**				
Non-hospitalized asymptomatic	9/162 (5.56)	Reference	Reference	
Non-hospitalized symptomatic	190/912 (20.83)	3.75 (1.92 – 7.32)	2.32 (1.15 – 4.70)	0.0193
Hospitalized (no oxygen)	105/614 (17.10)	3.08 (1.56 – 6.08)	3.96 (1.90 – 8.24)	0.0002
Hospitalized (oxygen therapy)	335/656 (51.07)	9.19 (4.74 – 17.82)	6.01 (2.94 – 12.29)	0.0000
Hospitalized (vent or intensive care unit)	787/1356 (58.04)	10.45 (5.42 – 20.15)	5.78 (2.87 – 11.66)	0.0000
**Variant**				
Beta	755/1268 (59.54)	Reference	Reference	
Delta	515/1590 (32.39)	0.54 (0.49 – 0.61)	0.93 (0.81 – 1.07)	0.3115
Omicron	156/842 (18.53)	0.31 (0.26 – 0.37)	0.45 (0.36 – 0.57)	0.0000
**COVID-19 vaccination status**				
Not vaccinated	398/1146 (34.73)	1.45 (1.21 – 1.73)	0.97 (0.80 – 1.18)	0.7544
Vaccinated before infection	175/731 (23.94)	Reference	Reference	
Vaccinated after infection	846/1804 (46.90)	1.96 (1.66 – 2.31)	0.91 (0.75 – 1.10)	0.3413
Unknown	7/19 (36.84)	1.54 (0.72 – 3.28)	0.81 (0.37 – 1.74)	0.5815

a IRR: adjusted incident risk ration; CI: confidence interval The following individual comorbidities had *P*-values <0.2 when assessed in univariate models: heart disease, asthma, cancer, rheumatological disorder, diabetes, hypertension and obesity. However, they were not significate in the multivariable model

## Discussion

We described a high prevalence of PCC at 6 months after the SARS-CoV-2 infection in a cohort of 3700 hospitalized and nonhospitalized participants in South Africa. Overall, 39% of participants experienced persistent symptoms at 6 months; 46.7% in hospitalized participants, and 18.5% in nonhospitalized participants. This differed by the variant period (lower prevalence of PCC among those infected during the Omicron period) and also by the severity of acute COVID-19 (higher prevalence of PCC with more severe acute COVID-19).

The prevalence of PCC in hospitalized South African individuals (47%) was similar to estimates reported by other studies at 6 months, 40% in Italy [32], 40% in China [33], 47% in Switzerland [34], 48% in Saudi Arabia [35], 50% in Russia [36], 57% in US [37], 60% in France [38], 61% in Norway [39], 68% in China [40], and 64% in a meta-analysis [41]. The differences in the prevalence of PCC by geographical region at 3 months have previously been reported, ranging between 31% in North America, 44% in Europe, and 51% in Asia [9].

Encouragingly, the prevalence of persistent symptoms in our cohort declined with successive follow-up periods, from 82% at 1 month to 53% at 3 months and 47% at 6 months for hospitalized participants and from 32% at 3 months to 19% at 6 months for nonhospitalized participants. A declining trend in persistent symptoms between 3 and 6 months has also been reported from 51% to 40% in a Chinese cohort [33] and from 68% to 60% in France [38]. Other studies with longer follow-up durations have reporting further declining trends at 12 months [36,42] and 24 months [4]. However, a significant proportion of participants in our study remained symptomatic at 6 months, representing a substantial potential individual and health system burden. Extrapolating the prevalence of PCC to the total hospitalized patients with COVID-19 (from hospital surveillance) and likely total infected individuals (from serosurveys), it is possible that over 250,000 hospitalized and 6.3 million nonhospitalized individuals had persistent symptoms at 6 months after SARS-CoV-2 infection in South Africa.

We also showed that the progression of PCC was not linear and was characterized by fluctuating trends over 6 months. This finding is consistent with the WHO's description of PCC characterized by a clinical progression, including a new onset of symptoms after initial recovery from an acute COVID-19 episode, persistent symptoms from the initial illness, or symptoms that fluctuate or relapse over time [1]. Studies have described the relapsing and remitting nature of PCC [37,43], but few have explored the nature of symptom progression in the detail provided by this study.

Fatigue was the most common symptom in our study at all time points and among all cohorts. COVID-19-induced fatigue can be defined as ‘a decrease in physical and/or mental performance that results from changes in central, psychological, and or/peripheral factors due to the COVID-19 disease’ [44]. Fatigue was reported to be the most debilitating PCC symptom and the main reason patients contacted a COVID-19 rehabilitation program [45].

The risk factors for PCC in our study included older age, female sex, non-Black race, the presence of a comorbidity (although individual comorbidities were not predictive), greater number of acute COVID-19 symptoms, hospitalization/ COVID-19 severity, and wave period. Other studies have reported on associations of PCC with female sex, >5 acute COVID-19 symptoms, comorbidities (pre-existing hypertension, chronic lung conditions, obesity, asthma), hospital admission, and severity of acute COVID-19 [6,[9], [10], [11],^13^], as well as oxygen treatment [6,46], steroid treatment [47], and treatment in the ICU [32]. Conflicting findings have been reported regarding age and race. Some studies reported increased risk with older age [[5], [6], [7],^10^,^13^,^34^,^48^]. Studies have identified higher risk of PCC among adults aged <70 years [27,49], among young adults [11,37,50], and among middle-aged adults aged 40-55 years [3,51]. Some studies reported increased risk of PCC for ethnic minority groups [5,48,52], whereas other studies concluded that race and ethnicity were not associated with PCC [53].

The risk of persistent symptoms at 6 months was higher as the severity of acute COVID-19 increased. Similar findings have been reported of a higher burden of PCC among those hospitalized compared with nonhospitalized [54] and among those with severe acute COVID-19 in systematic reviews [6,55] and cohort studies [56]. The mechanisms for increased risk of PCC with severity include more severe immune response and cytokine storm, resulting in more organ damage, as well as more aggressive treatment [57].

We described a lower risk of persistent symptoms among participants infected in the Omicron wave. Similar findings have been reported in other studies with shorter follow-up duration [9,[14], [15], [16]]. It, however, remains unclear whether this reduction in PCC is related to the Omicron variant itself or a result of the effect of a previous immunity from vaccination or natural infection, which resulted in milder acute COVID-19 infection.

A few studies have suggested that COVID-19 vaccination reduces the likelihood of PCC [17], [18], [19] and triggered an improvement in symptoms [58,59]. The mechanisms suggested for this include the destruction of residual viral reservoir by the antibody response and a “reset” of autoimmune dysregulation [19,46]. In our study, there was no difference in persistent symptoms in vaccinated and unvaccinated participants. However, it is important to note that our study did not record objective vaccination status, details of partial versus complete vaccination, precise temporality to infection, or serological confirmation of immunity.

Finally, our study also demonstrated the significant impact of PCC on quality of life; however, the quality of life improved for all domains over time. The quality of life domains that were still significantly affected at 6 months were fatigue (38%), disability (24%), and pain/discomfort (16%). In a systematic review, all studies with follow-up of 3-6 months after COVID-19 demonstrated worsened mobility, self-care, usual activities, pain/discomfort, and anxiety/depression, resulting in loss of independence [60]. Systematic reviews reported activity impairment, disability, and disruption in work life [6,8,61].

### Strengths and limitations

Our study was a large, nationally representative longitudinal cohort study and, to the best of our knowledge, the first of its kind in South Africa and sub-Saharan Africa. As part of the global ISARIC collaboration, we used standardized and validated tools, which allows comparison across participating countries.

The study had several limitations. First, we did not include controls with respiratory infection other than COVID-19 to understand the effect of COVID-19 on continuing morbidity. Second, all participants were enrolled through a telephone assessment, limiting the enrollment to those who had a telephone number recorded. Third, there is a possibility of response bias, and participants who had symptoms might have been more likely to participate than those who did not. These limitations are evident in the comparison of the study participants with the population of SARS-CoV-2 cases and COVID-19 hospitalizations, with study participants being younger and more likely from the private sector (therefore, more likely to have a contact number and to respond) and more study participants with comorbidities (therefore more likely to participate than those with no persistent symptoms). The high retention and minimal loss to follow-up in our study strengthens our findings (retention rate was 78.8% and 79.5% for hospitalized and nonhospitalized participants, respectively, at 6 months). Recall bias is possible because participants were recruited at 1 month or 3 months and asked to report their symptoms during the acute COVID-19 infection. Some bias could also have been introduced due to self-reporting of comorbidities and vaccination status. Finally, the lack of objective metrics to assess symptoms might affect reporting, which is a limitation of all PCC studies [34,62], and future work should focus on developing objective assessments and examining their correlation with self-reported measures.

## Conclusion

The study revealed a high prevalence of PCC among South African participants at 6 months but encouragingly showed a decreasing prevalence of persistent symptoms and impact on quality of life over time. The study improves our understanding of the nature and evolution of PCC, the risk factors associated with PCC in the South African context, and the differences in PCC by circulating SARS-CoV-2 variants of concern. The finding of decreased PCC at 6 months among participants infected during the Omicron wave is important because it may suggest decreasing rates of PCC with greater population immunity.

These findings have serious implications for low-middle-income countries, which have resource-constrained health care systems that may now need to also establish postacute care services in settings where physical, cognitive, and mental health disabilities often go under-recognized [50]. Low-middle-income countries also do not generally have social safety nets, and the impact of chronic sequelae on the workforce and on families’ livelihoods remain a concern.

The evidence generated by this study will help to inform the national public health response to PCC, which should include sensitizing and training health care workers on managing patients with PCC, updating clinical guidelines, and establishing multidisciplinary health services at least in large public hospitals. These findings will also inform the development of additional patient support forums and educational material for patients with PCC. The study continues to follow-up participants and will report on trends at the final survey to be conducted at 12 months.

## Declaration of competing interest

The authors have no competing interests to declare.

## Appendix Supplementary materials

Image, application 1
